# Subtle changes on electrocardiogram in severe patients with COVID-19 may be predictors of treatment outcome

**DOI:** 10.3389/frai.2025.1561079

**Published:** 2025-03-12

**Authors:** Illya Chaikovsky, Dmytro Dziuba, Olga Kryvova, Katerina Marushko, Julia Vakulenko, Kyrylo Malakhov, Оleg Loskutov

**Affiliations:** ^1^Department of Anaesthesiology and Intensive Care, Shupyk National Healthcare University, Kyiv, Ukraine; ^2^Department of Contactless Control Systems, V.M. Glushkov Institute of Cybernetics of the National Academy of Sciences, Kyiv, Ukraine; ^3^Department of Medical Information Technologies, International Research and Training Center of the National Academy of Sciences, Kyiv, Ukraine; ^4^Department of Anaesthesiology and Intensive Care for Infectious Diseases, Kyiv City Clinical Hospital No. 4, Kyiv, Ukraine; ^5^Microprocessor Technology Lab, V.M. Glushkov Institute of Cybernetics of the National Academy of Sciences, Kyiv, Ukraine

**Keywords:** electrocardiography, myocardial injury, severity, mortality, COVID-19, machine learning, cloud computing

## Abstract

**Background:**

Two years after the COVID-19 pandemic, it became known that one of the complications of this disease is myocardial injury. Electrocardiography (ECG) and cardiac biomarkers play a vital role in the early detection of cardiovascular complications and risk stratification. The study aimed to investigate the value of a new electrocardiographic metric for detecting minor myocardial injury in patients during COVID-19 treatment.

**Methods:**

The study was conducted in 2021. A group of 26 patients with verified COVID-19 diagnosis admitted to the intensive care unit for infectious diseases was examined. The severity of a patient’s condition was calculated using the NEWS score. The digital ECGs were repeatedly recorded (at the beginning and 2–4 times during the treatment). A total of 240 primary and composite ECG parameters were analyzed for each electrocardiogram. Among these patients, 6 patients died during treatment. Cluster analysis was used to identify subgroups of patients that differed significantly in terms of disease severity (NEWS), SрО_2_ and integral ECG index (an indicator of the state of the cardiovascular system).

**Results:**

Using analysis of variance (ANOVA repeated measures), a statistical assessment of changes of indicators in subgroups at the end of treatment was given. These subgroup differences persisted at the end of the treatment. To identify potential predictors of mortality, critical clinical and ECG parameters of surviving (S) and non-surviving patients (D) were compared using parametric and non-parametric statistical tests. A decision tree model to classify survival in patients with COVID-19 was constructed based on partial ECG parameters and NEWS score.

**Conclusion:**

A comparison of potential mortality predictors showed no significant differences in vital signs between survivors and non-survivors at the beginning of treatment. A set of ECG parameters was identified that were significantly associated with treatment outcomes and may be predictors of COVID-19 mortality: T-wave morphology (SVD), Q-wave amplitude, and R-wave amplitude (lead I).

## 1 Introduction

Experience with the pandemic has shown that the disease can pose a severe threat to the lives of patients. The main danger of the disease is acute respiratory syndrome and lung injury. However, patients may experience damage to other organs and systems: the cardiovascular system, the immune system, the liver and the kidneys. Myocardial injury occurred in at least 10% of unselected COVID-19 cases and up to 41% in critically ill patients or those with comorbidities (Cameli et al., 2020).

In the survivors, the majority showed long-term symptoms, now often referred to as long COVID-19 (Garg et al., 2021; Crook et al., 2021). One of the critical long-term clinical consequences of COVID-19 seems to be myocardial injury (Guzik et al., 2020; Italia et al., 2021; Akhmerov and Marbán, 2020).

Signs and symptoms of possible myocardial injury after COVID-19 may include severe fatigue, palpitations, chest pain, shortness of breath, postural orthostatic tachycardia syndrome (POTS) due to neurologic disturbances, post-exertional fatigue, and higher troponin levels (Lovell et al., 2022; Bandyopadhyay et al., 2020; Xie et al., 2022; Matsumori et al., 2022).

In addition, COVID-19 appears to cause severe myocarditis. It can affect the myocardium and pericardium, causing severe fatigue without other apparent symptoms (Lovell et al., 2022). Diagnosis of myocarditis is relatively inaccurate because both tests and diagnostic protocols lack accuracy. Some reports showed that symptoms persisted for an average of 47 days before being diagnosed by cardiac magnetic resonance (CMR) imaging (Ojha et al., 2021).

Therefore, it is extremely important to identify critical factors for assessing COVID-19 severity, predicting treatment outcomes, and optimizing individual treatment strategies (Izcovich et al., 2020; Fathi et al., 2021). It is known that 49 variables can provide valuable prognostic information about mortality and disease severity in patients with COVID-19 (Izcovich et al., 2020).

Numerous studies have confirmed that cardiac (Mueller et al., 2021) and other biomarkers may reflect cardiovascular injury and inflammation in COVID-19 and are strongly associated with poor prognosis and mortality (Battaglini et al., 2022; Wang et al., 2020). In addition, some electrocardiographic (Bergamaschi et al., 2021) and echocardiographic alterations (Long et al., 2021) appear to have prognostic implications for patients with COVID-19.

Several prognostic models have been developed to assess disease severity in patients with COVID-19 and predict mortality (Bertsimas et al., 2020; Pourhomayoun and Shakibi, 2021; Liang et al., 2020; Zhou et al., 2021; Yan et al., 2020; Kang et al., 2021).

Such classification models have usually been developed using various machine learning (ML) algorithms. For example, one neural network model has demonstrated 93% accuracy in predicting mortality based on patients’ physiological status, symptoms, and demographic information (Pourhomayoun and Shakibi, 2021).

A multivariable logistic regression model and an online risk calculator based on 10 clinical indicators were proposed to predict critical illness development among hospitalized patients with COVID-19 (Liang et al., 2020). A support vector machine (SVM) model based on 11 routine clinical parameters was developed to assess the severity of COVID-19 patients (Zhou et al., 2021).

An interpretable mortality prediction model for COVID-19 patients was proposed by Yan et al. (2020) where the XGBoost ML algorithm was used to select predictors. The interpretable decision tree and the decision rule for 3 biomarkers that predict the survival of individual patients with more than 90% accuracy were obtained.

It should be noted that in one of the ML models for predicting the severity of COVID disease, among the 33 analyzed signs and indicators, there was the cardiac functional grading (according to New York Heart Association functional classification) (Kang et al., 2021). However, this cardiac indicator was excluded from the model because of its weak positive correlation with the severity of COVID-19.

In this context, the advanced analysis of ECG is highly demanded. This is especially true for patients with a normal or slightly changed electrocardiogram, i.e., if the analysis did not reveal any “major” category according to the, for example, Minnesota coding system. The only way to increase the diagnostic value of ECG examination is to develop proper information technology (IT)—a combination of up-to-date methods and equipment bound into a chain that provides collection, storage, pre-processing, interpretation, conclusion and dissemination of information (Chaikovsky, 2020).

At the same time, the advancement of diagnostic methods, especially instrumental ones (i.e., methods of functional diagnostics), primarily entails a constant increase of their “distributive capacity”—the ability to detect more and more and subtler changes in the function examined by one method or another. Such opportunities emerge due to progress in technical measurement tools of a specific function and even more due to the development of informational technologies. In other words, due to the creation of new metrics—numerical parameters using which one can assess the aspects of the functioning of various human organs and systems that were inaccessible before.

As a result, new ways of improving the diagnostic accuracy of a particular method within its traditional application scenarios are discovered. Additionally, familiar methods find unconventional uses in new areas.

Everything mentioned above fully applies to the new informational technologies for cardiac electrical activity assessment developed at V.M. Glushkov Institute of Cybernetics of the National Academy of Sciences of Ukraine.

The main goal set by the developers in this context was to make any electrocardiography informative. Routine ECG analysis is based on specific ECG syndromes or phenomena defined within one of the existing visual ECG analysis algorithms. However, in most cases, no ECG syndrome can be identified during the analysis of an individual electrocardiogram, at least not one that reflects cardiac pathology, i.e., belongs to the “major” category according to the Minnesota coding system, for example. During the routine analysis, one is forced to assign a single class to all these electrocardiograms—electrocardiograms with no primary ECG syndrome identified. However, the question arises: are all these electrocardiograms the same in terms of their relative “distance” to the “ideal” electrocardiogram of a healthy human? They are not. Depending on the myocardial condition, this “distance” can be further or closer. Moreover, there is a reasonable hypothesis that this “distance” reflects the likelihood of serious cardiovascular events. This is where routine analysis of an electrocardiogram is uninformative.

That is why the Universal Scoring system method and software for ECG scaling that can provide the quantitative evaluation of the slightest changes in ECG signal were developed (Chaikovsky, 2020; Chaykovskyy et al., 2019). This approach is based on, first of all, measuring the maximum number of ECG parameters and heart rate variability and, secondly, on positioning each parameter value on a scale between the absolute norm and extreme pathology. The suggested approach follows a popular Z-scoring ideology, where quantitative, usually point-based assessment of test results is determined using a unique scale containing data about intra-group test results variation. To calculate the Z-score mean, the test value of the group and its standard deviation are needed (Colan, 2013).

This study aimed to investigate the value of a new electrocardiographic metric for detecting subtle myocardial injury in patients during COVID-19 treatment. And also, to test the hypothesis about the prognostic value of myocardial injury on the treatment outcome.

## 2 Materials and methods

### 2.1 Study design and patient characteristics

The study was conducted in 2021. A total of 26 patients with confirmed COVID-19 were monitored while on treatment in the intensive care unit (ICU) of the Kyiv Clinical Hospital #4. The hospitalization duration ranged from 5 to 27 days. All the patients were initially in a severe condition.

The vital signs were documented to evaluate the course of the illness: heart rate, blood oxygen saturation, blood pressure, body temperature, and respiration rate. Based on them, the severity of a patient’s condition was calculated using a widely accepted NEWS score (Wang et al., 2020). Severe COVID-19 condition was defined as meeting NEWS aggregate score of 7 or over.

**Figure 1:**
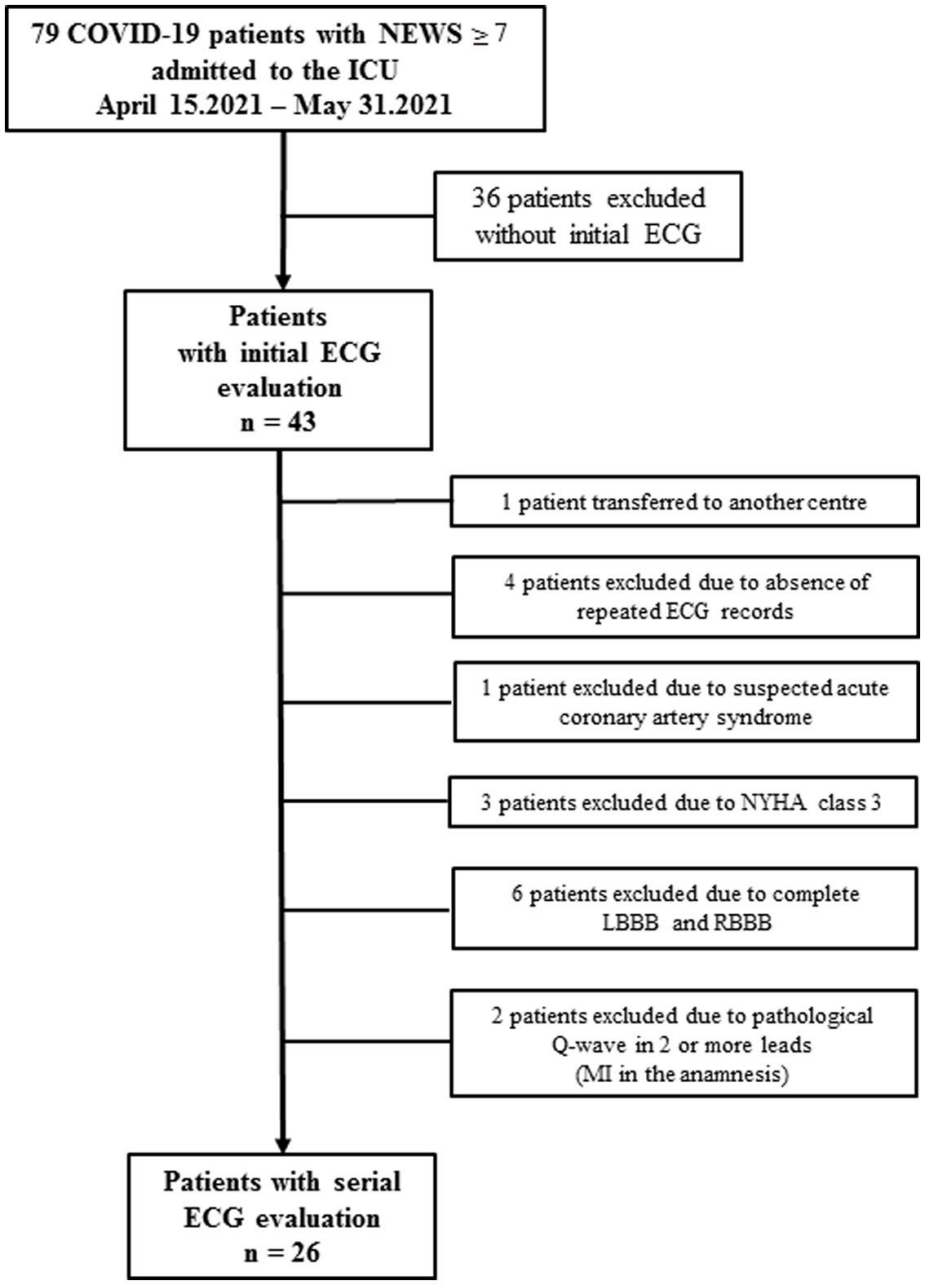
Flow-chart of the enrollment of COVID-19 patients with serial ECG evaluations.

The process of patients’ enrollment is presented on Figure 1.

Most patients had a history of chronic diseases, first of all CVD, but these diseases were in remission. Thus, only patients with no signs of instability in relation to heart disease and no gross changes in the electrocardiogram in accordance with Minnesota coding were included in the study. Based on this logic, as shown in Figure 1, four patients were excluded due to instability of their conditions in the context of comorbid heart diseases, and eight other patients were excluded because they had major pathological changes on the ECG according to Minnesota coding.

In 26 patients, an ECG in 12 leads by serial digital ECG device (Solvaig Ltd., Ukraine) was repeatedly recorded (at the beginning and several times during the treatment—from 2 to 4 times). Among these patients, six patients died during the treatment. The main characteristics of the patient’s condition were recorded several times. The integral indicators were used to calculate the patient’s severity according to the NEWS scale (National Early Warning Score) (Baker et al., 2021) and SAPS II (The Simplified Acute Physiology II Scale) (Allyn et al., 2016). А demographic and anthropometric values, clinical parameters and ICU characteristics are presented in Table 1.

**Table 1:** Patient characteristics at ICU admission.

Parameter	Value
Age (years)	63 ± 14.1
Sex (female)	10 (38.47%)
Sex (male)	16 (61.53%)
Weight (kg)	80.4 ± 16.65
Length (cm)	166.5 ± 9.09
BMI (kg/m^2^)	28.48 ± 6.5
Hypertension	22 (84%)
Diabetes mellitus	9 (34%)
Ischemic heart disease	18 (69%)
Heart failure	11 (42%)
Pulmonary embolism	11 (42%)
Pulmonary hypertension	26 (100%)
Malignant disease	3 (11.5%)
Liver failure	2 (7.6%)
Vascular disease	4 (15.3%)
Days with symptoms at ICU admission	11.5 (5–27)
SAPS II	25 (9–35)
NEWS	7.34 (3–10)

Data are presented as (mean ± standard deviation), median (interquartile range) and numbers (percentages). ICU: Intensive care unit. BMI, body mass index; SAPS II, Simplified Acute Physiology Score II; NEWS, National Early Warning Score.

### 2.2 Statistical analysis

Data are presented as means ± standard deviation (SD) or median (interquartile range, IQR) for continuous variables, based on normality and as percentages for categorical variables. A two-sample *t*-test compared the baseline characteristics of subjects within each group with unequal variances for continuous variables. Mann–Whitney *U* test was performed for variables that were not normally distributed. Two-tailed *p* < 0.05 was considered statistically significant.

The expectation–maximization (EM) clustering algorithm with 10-fold cross-validation was used to identify homogeneous groups. Homogenous groups were formed based on disease severity and integral index of patients at the beginning of treatment. As a result, two subgroups were identified that were significantly different in the severity of the disease and the integral indicator of the state of the cardiovascular system of patients at the start of treatment. Repeated measures ANOVA was used to evaluate statistical differences in the main clinical parameters in these subgroups at the beginning and the end of treatment.

We used machine learning algorithms (CART) such as Decision Trees to construct a model for classifying patient mortality. Statistical analysis was performed using Statistica 12.0 software.

## 3 Results

### 3.1 Correlation and cluster analysis, changes of integral parameters in the course of treatment

Previous studies have shown heterogeneity in clinical manifestations, severity and outcomes in COVID-19 patients. Our task was to study the heterogeneity of patients, taking into account the vital signs, the severity of the disease, and the state of the cardiovascular system (CVS). In addition, it was necessary to determine the influence of these factors on the treatment outcome.

We calculated correlation coefficients for all monitoring data to study the relationship between the CVS state’s integral index and patients’ vital signs during treatment. Mean values and Spearman correlation coefficients for the vital signs and the composite index of ECG U-score are shown in Table 2.

**Table 2:** Mean values and Spearman’s correlation coefficients for vital signs and U-score composite ECG index.

Variables	Mean ± SD	HR	SрО_2_	*t*	NEWS	SAPS II	U-score
HR (heart rate)	80.05 ± 11.98	1.00	−0.24*	−0.10	0.10	0.06	−0.11
SрО_2_ (blood oxygen saturation)	86.46 ± 4.76	−0.24*	1.00	−0.24*	−0.64*	−0.12	0.28*
Body temperature (t°C)	37.17 ± 0.66	−0.10	−0.24*	1.00	0.46*	−0.52	−0.22*
NEWS score	6.14 ± 2.24	0.10	−0.64*	0.46*	1.00	−0.25	−0.25*
SAPS II	23.5 ± 6.14	0.06	−0.12	−0.52*	−0.25	1.00	−0.17
U-score	59.49 ± 10.66	−0.11	0.28*	−0.22*	−0.25*	−0.17	1.00

SD, standard deviation, *p < 0.05.

As one can see, a weak but statistically significant correlation exists between U-score composite ECG index and blood oxygen saturation, body temperature and NEWS score (in the last two cases - negative correlation).

Table 3 shows the distribution of patients according to the NEWS score, indicating the heterogeneity group of patients at the beginning of treatment.

**Table 3:** The distribution of patient severity score at the beginning of treatment.

NEWS score	Number of cases	%
3	2	6.25
5	2	6.25
6	2	6.25
7	8	25.00
8	13	40.63
9	3	9.38
10	2	6.25

To identify homogeneous subgroups (clusters), the sample of 26 patients was analyzed using EM cluster analysis with 10-fold cross-validation.

Hence, the problem of choosing the best parameters for clustering arises. In the general case, the task of selecting features for clustering consists of choosing the “best” set of features that helps to identify natural clusters according to the criterion. In our case, the criterion is the treatment outcome. Preliminary, feature dimensionality reduction methods were used to collapse features, in other words, to form composite features. Based on this logic, the electrocardiographic composite score called U-score, as well as composite scores NEWS and SAPS II for assessing the patient’s condition in the intensive care unit, were primarily selected as features for clustering. Then, SAPS II was rejected due to its minimal variations during treatment. In addition, the SpO_2_ value was chosen as a clustering parameter, since this independent feature is the most important vital sign in assessing the condition of patients with respiratory distress. Other vital signs such as HR, body temperature, as well age was also preliminarily investigated as potential parameters for clustering, but were rejected because the clusters resulting from the use of these parameters were clearly unrelated to the target criterion.

As a result, the NEWS score (as a categorial variable), SрО2 and U-score composite ECG index at the beginning of treatment have been taken for clusterization. NEWS and U-score were taken for clustering as the most integral indexes, and SpO_2_ - since this is the vital sign for patients in the ISU for COVID-19.

As a result, two subgroups significantly differing from each other in SpO_2_, NEWS score and U-score values were identified among these patients:

Cluster 1 included 19 patients with mean NEWS = 7.1, SрО_2_ = 84.3, U-score = 60.5.

Cluster 2 included 7 patients with mean NEWS = 8.3, SрО_2_ = 78.0, U-score = 49.8.

**Figure 2:**
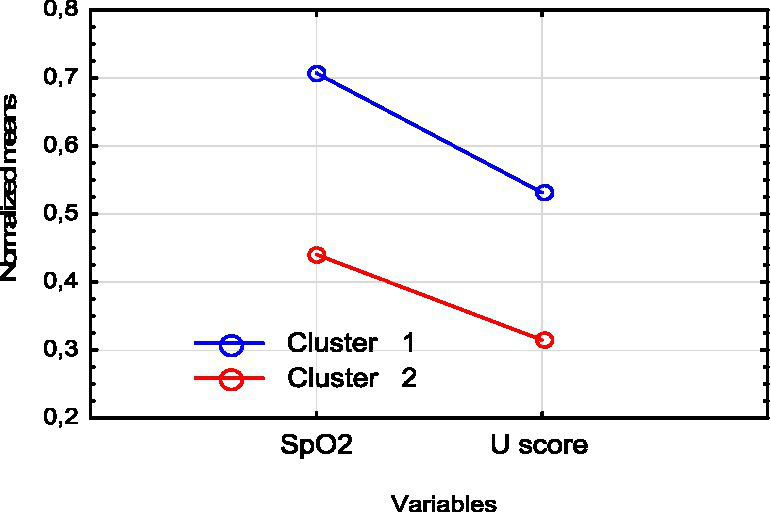
Normalized means SpO_2_ and U-score in cluster 1 and cluster 2.

A diagram of standardized values of SpO_2_ and U-score is shown in Figure 2. As we can see, at the beginning of treatment, patients from cluster 1 have higher levels of oxygen and an integral ECG index compared to cluster 2. Сluster 2 (subgroup 2) is characterized by a combination of greater severity with low oxygen level and lower U-score (integral ECG index level).

The average values of vital signs in the identified subgroups are shown in Table 4.

**Table 4:** Comparison of vital signs in cluster 1 and cluster 2 (*t*-test).

Vital signs	Cluster 1 (*n* = 19) Mean ± SD	Cluster 2 (*n* = 7) Mean ± SD	*p*-value
Age	59.63 ± 10.23	71.43 ± 18.48	0.047
BR (breathing rate)	23.84 ± 3.20	24.86 ± 2.91	0.470
HR	82.05 ± 15.06	88.29 ± 11.35	0.331
SpO_2_	84.37 ± 2.48	78.00 ± 4.47	0.0001
Body temperature	37.46 ± 0.81	37.79 ± 0.73	0.358
NEWS	7.16 ± 1.77	8.29 ± 1.25	0.136
SAPS II	23.19 ± 5.75	25.67 ± 7.37	0.413
U-score	60.05 ± 11.87	49.86 ± 7.90	0.046

SD, standard deviation; n, number of patients.

In addition, the subgroup with a more severe course of the disease (cluster 2) significantly differs in the age of patients. In subgroup 2, patients are older but do not differ in physiological severity. They do not have a significant difference in the indicator of physiological severity SAPS II.

**Figure 3:**
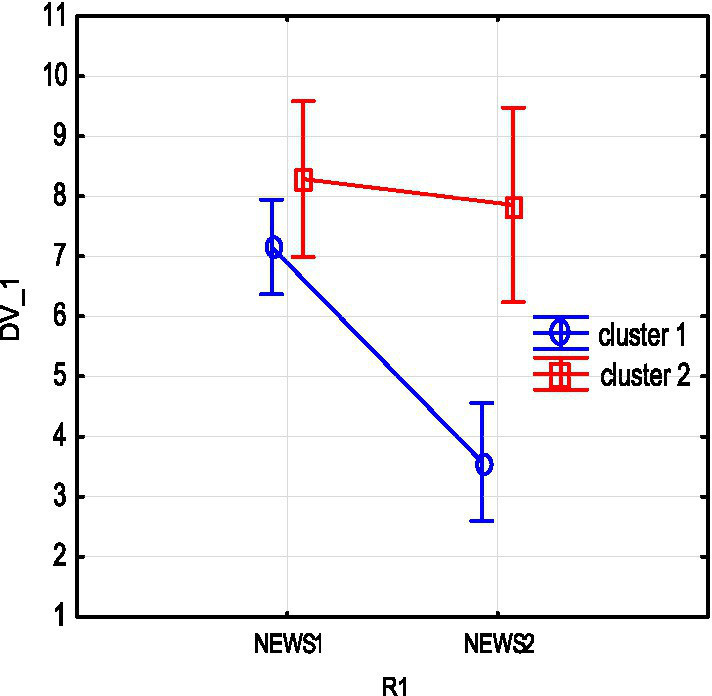
NEWS severity score changes due to treatment in clusters 1 and 2.

**Figure 4:**
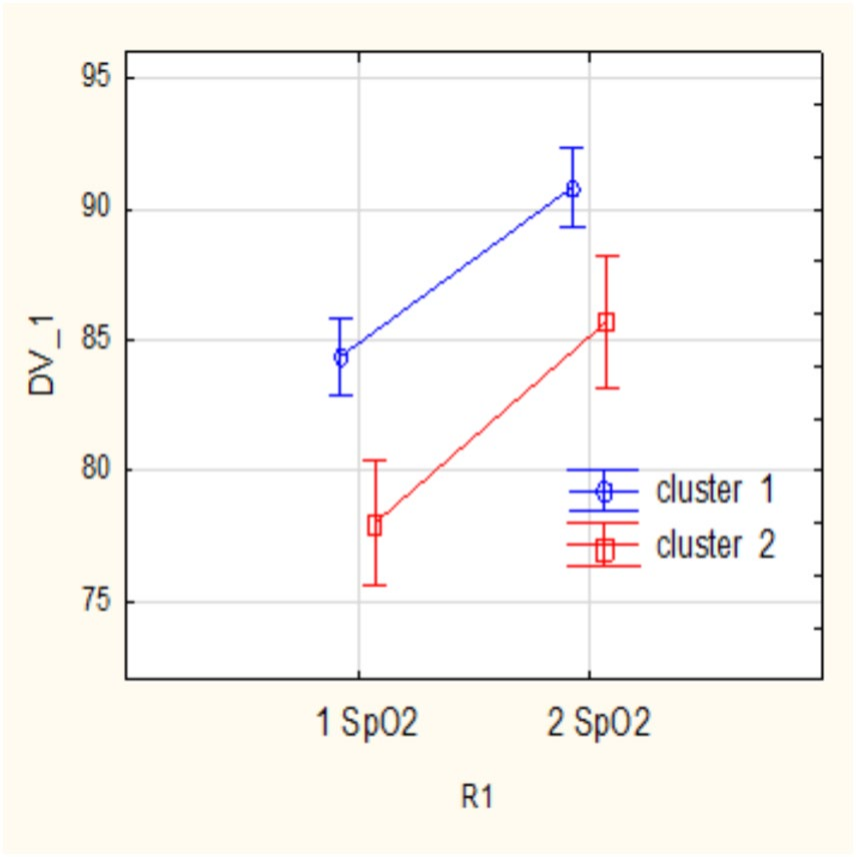
SpO_2_ changes due to treatment in clusters 1 and 2.

**Figure 5:**
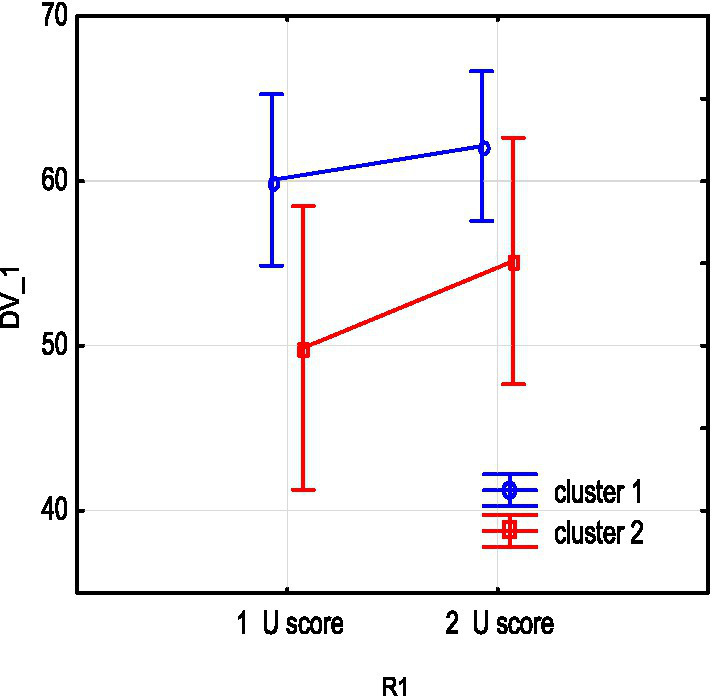
U-score integral ECG index changes due to treatment in clusters 1 and 2.

We studied the dynamics of the abovementioned main parameters (SpO_2_, NEWS, U-score) in two clusters throughout treatment using a repeated measures analysis of variance (RepANOVA). The changes of these parameters at the beginning (1) and by the end (2) of treatment are shown in Figures 3–5.

As shown from the figures above, both subgroups show a decrease in NEWS severity score and an increase in SpO_2_ as a result of treatment, and these changes are statistically significant. The impact of therapy on main parameters in subgroups can be assessed by partial effect sizes (partial eta-squared, $\eta_{p}^{2}$ ). The effect of increasing oxygen in each cluster is significant: cluster 1 R1 SpO_2_ $\eta_{p}^{2}$ = 0.73, *p* = 0.00001; cluster 2 $\eta_{p}^{2}$ = 0.72, *p* = 0.007.

In addition, the dynamics of severity reduction in the 1st subgroup is more pronounced. Sub NEWS partial eta-squared = $\eta_{p}^{2}$ = 0.7, *p* = 0.0001. Note that a decrease in the mean NEWS is not statistically significant in subgroup 2 (cluster 2 with severe baseline).

In addition, the dynamics of severity reduction in the 1st subgroup is more pronounced. Sub NEWS partial eta-squared = $\eta_{p}^{2}$ = 0.7, *p* = 0.0001. Note that a decrease in the mean NEWS is not statistically significant in subgroup 2 (cluster 2 with severe baseline).

The U-score composite index has a positive tendency to increase. However, the wide dispersion observed indicates a heterogeneity of U-score changes. Note that in subgroup 2, with low initial levels of integral indicator and oxygen, the part of unfavorable outcomes (ratio deceased/survivors) is 3 out of 7, >3 out of 19 in subgroup 1.

### 3.2 Comparison of vital signs and ECG indicators in two groups by the outcome of treatment

Next, we studied the differences between patient groups formed according to treatment outcomes. The study group of 26 patients consisted of two classes according to the treatment outcome: 20 survivors and 6 non-survivors. Clinical data and ECG parameters at the beginning and end of treatment were compared between survivors (S) and non-survivors (D).

At the beginning of treatment, there were no significant differences between groups S and D in the vital signs (SAPS II, SpO_2_, NEWS score and U-score), except for the patient’s age and body temperature (Table 5).

**Table 5:** Comparison of mean vital signs between survivors and non-survivors (*t*-test).

ECG indicators	Survivors (*n* = 20) Mean ± SD	Non-survivors (*n* = 6) Mean ± SD	*p*-value
SAPS II	23.61 ± 5.91	25.00 ±	0.693
Age	59.30 ± 11.06	74.50 ±	0.013
SpO_2_	82.95 ± 4.16	81.67 ±	0.521
Body temperature	37.47 ± 0.83	37.80 ± 0.63	0.023
NEWS	7.30 ± 1.89	8.00 ± 0.63	0.388
HR	77.25 ± 20.67	78.50 ± 13.91	0.891
U-score	58.10 ± 10.83	54.67 ± 15.23	0.541

Then, an analysis was performed to identify any statistically significant differences among all partial ECG indicators constituting the U-score. The results of comparisons of ECG indicators in these groups at the beginning of treatment according to the *t*-test are shown in Table 6 and according to the Mann–Whitney *U* test in Table 7.

**Table 6:** Mean ECG indicators for survivors and non-survivors at the beginning of treatment (*t*-test).

ECG indicators	Survivors (*n* = 20) Mean ± SD	Non-survivors (*n* = 6) Mean ± SD	*p*-value
Т-wave morphology (SVD)	28.95 ± 49.16	98.67 ± 84.44	0.017
P/QRS integrals ratio	0.151 ± 0.09	0.062 ± 0.05	0.039
Integral of QRS complex (I)	0.023 ± 0.01	0.037 ± 0.02	0.025
U-score based composite index of cardiovascular events risk assessment	60.4 ± 21.30	38.3 ± 22.40	0.037
R-wave amplitude (μV) (lead I)	593.1 ± 231.00	861.8 ± 356.08	0.036
Q-wave amplitude (μV) (lead I)	−18.40 ± 29.19	−32.67 ± 43.83	0.358
R-wave amplitude (μV) (lead II)	412.85 ± 210.75	313.00 ± 263.49	0.345

n, number of patients.

**Table 7:** Chi-square statistics: outcomes—vital signs.

Vital signs (at the beginning of treatment)	Chi-square	*p*-value
Age	13.09	0.109
SAPS II	11.02	0.201
Body temperature	5.95	0.428
HR	5.15	0.741
BR	2.90	0.714
SpO_2_	5.11	0.647
NEWS	3.211	0.782

At the beginning of the treatment, statistically significant differences were observed among the following parameter values:

1. T-wave morphology (SVD);
2. P/QRS integrals ratio;
3. U-score based composite index of cardiovascular events risk;
4. R-wave amplitude (μV) (lead I);
5. Integral of QRS complex (lead I).

By the end of the treatment, statistically significant differences were observed, in addition to the severity of the disease, among the same ECG parameters and also in one of the fundamental heart rate variability indicators, RMSSD, which represents parasympathetic nervous system activity.

### 3.3 Vital signs and ECG indicators as treatment outcome predictors

In the last step of statistical treatment, we explored which parameters had the highest correlation with treatment outcomes using vital signs and the entire ECG and HRV dataset. We used univariate feature selection for classification based on the Chi-square statistical test (Tables 7, 8). Variables with higher Chi-square statistical values are then selected as predictors for classification.

**Table 8:** ECG indicators—treatment outcome predictors.

Treatment outcome predictors (at the beginning of treatment)	Chi-square	*p*-value
R-wave amplitude (mV) (lead II)	18.01	0.011
Т-wave morphology (SVD)	9.56	0.043
Q-wave amplitude (μV) (lead I)	12.26	0.052
Shift of the ST segment after 0.08 s after point J (mV) (lead I)	13.23	0.104
R-wave amplitude (μV) (lead I)	11.17	0.135
U-score based composite index of cardiovascular events risk	8.29	0.141

As follows from Table 8, vital signs at the beginning of treatment cannot be selected as treatment outcome predictors.

ECG indicators that significantly correlate with the treatment outcome class attribute and previously identified features (independent-group *t*-test) listed in Table 9 ranged *p*-value.

**Table 9:** The contribution (rank) of parameters to the survival classification model.

Parameters	Variable rank	Importance
Т-wave morphology (SVD)	100	1.000
R-wave amplitude (μV) (lead II)	98	0.977
Q-wave amplitude (μV) (lead I)	28	0.278

After testing the features with several statistics, we applied the wrapping method to the extracted features. With this approach, we evaluate the effectiveness of a subset of features, considering the final result of the applied learning algorithm (increase in accuracy when solving the classification problem).

**Figure 6:**
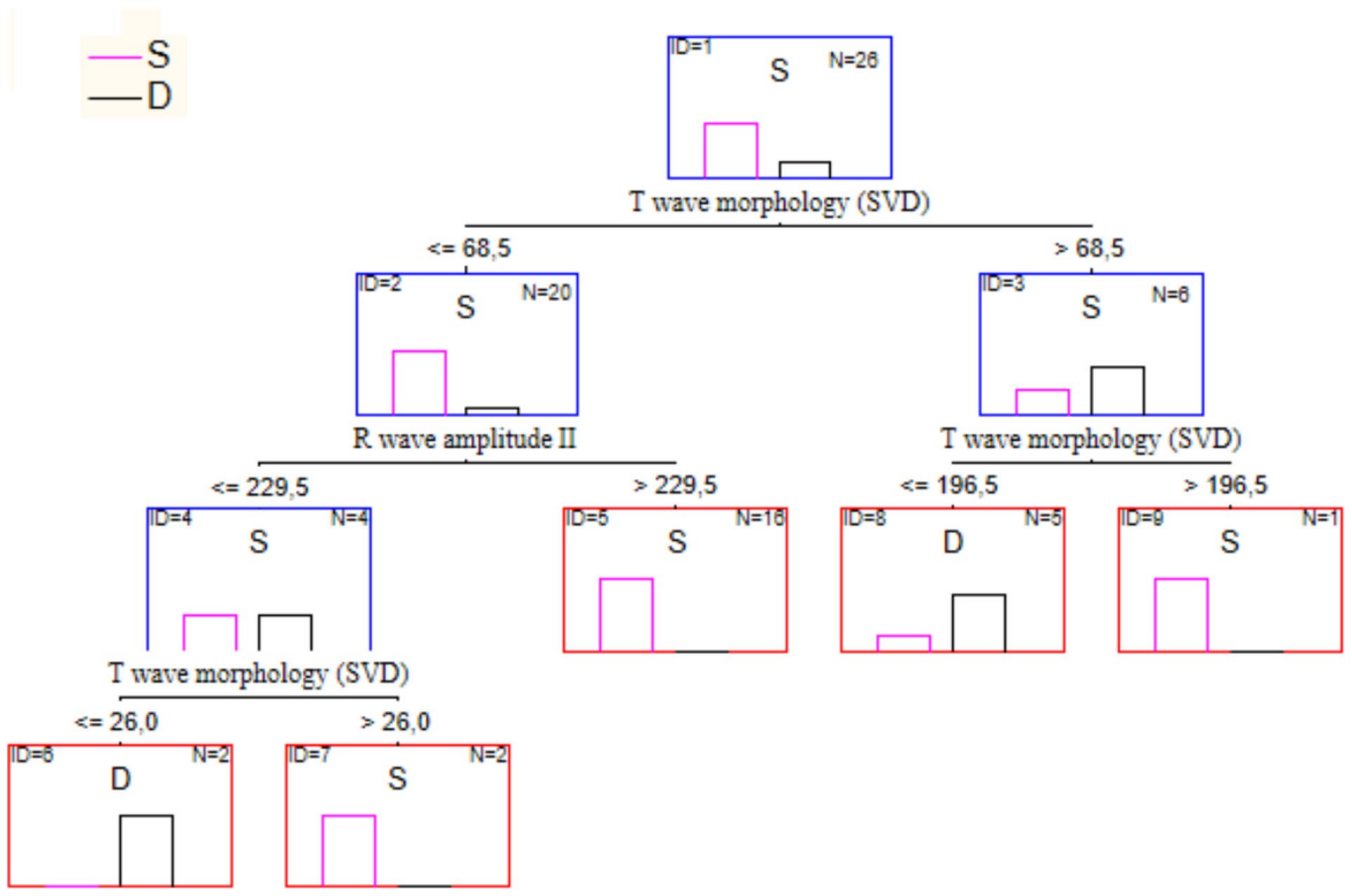
The optimal decision tree for the treatment outcome prognosis.

We used the CART machine learning algorithm (decision tree method) with 10-fold cross-validation to divide patients into two classes (S, D) according to the tested feature sets. As a result, a set of features with the highest classification accuracy (one classification error) was obtained. Figure 6 shows the optimal classification tree built based on three features.

Contribution of 3 ECG parameters to the resulting rules listed in Table 9. Classification accuracy—96%. One of the recovered patients was erroneously classified as deceased.

**Figure 7:**
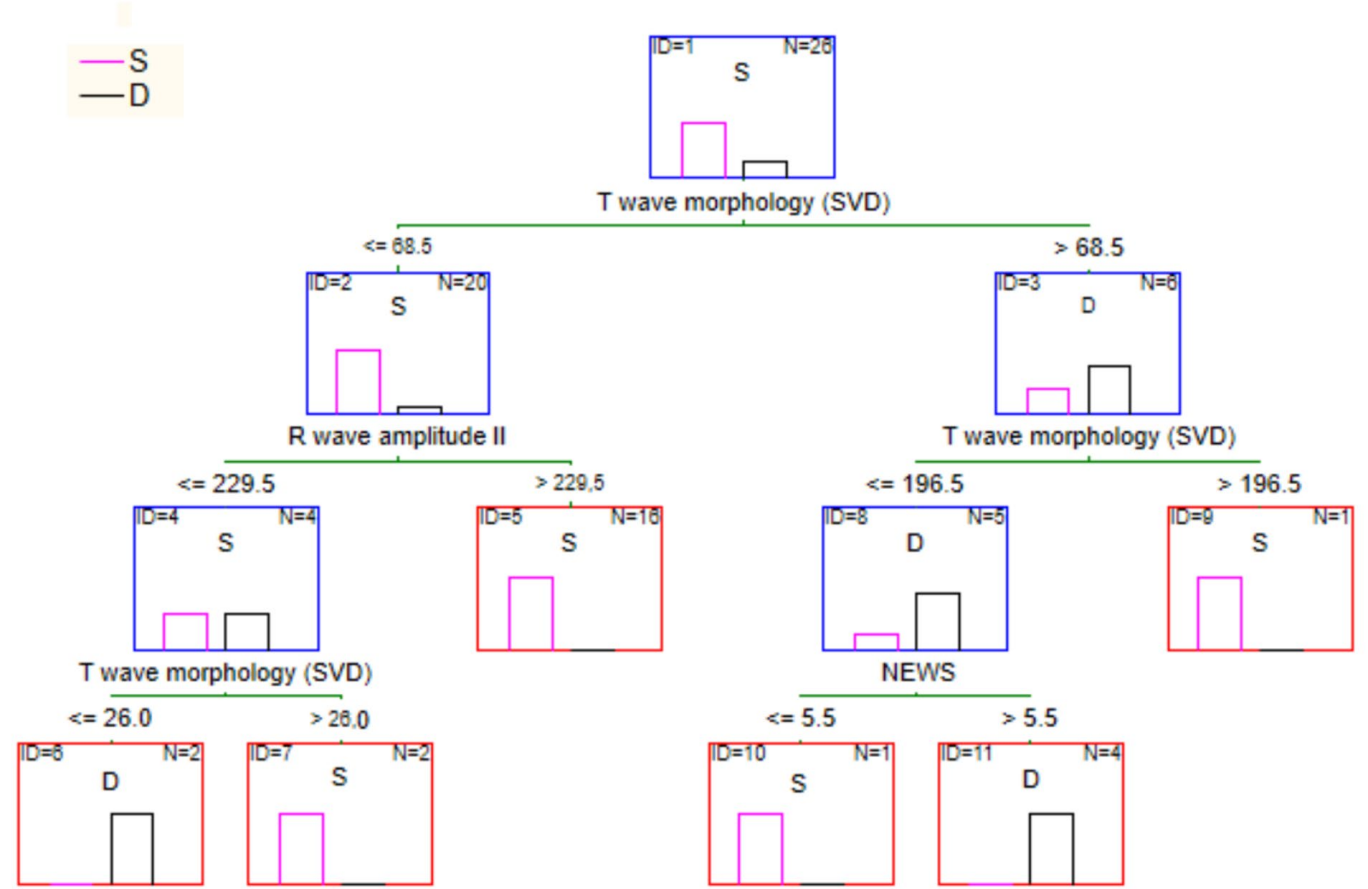
Full tree for the survival classification according to ECG parameters and NEWS score.

If one builds a tree using both parameters above and an additional attribute, NEWS score value, the result will be as shown in Figure 7. This tree has the same structure as the previous one. Still, it is right branch has an additional split determined by the NEWS score condition. In this case, the classification accuracy on the training set was 100%.

## 4 Discussion

This study and our previous works showed that the combination of ECG and HRV parameters has the best diagnostic value (Chaikovsky et al., 2019; Chaikovsky et al., 2020; Apykhtin et al., 2018; Patrick Neary et al., 2014; Clarke et al., 2020; Chaikovsky et al., 2020).

Changes in individual ECG and HRV parameters demonstrate only certain aspects of the examined phenomenon. Moreover, they can occur in opposite directions. Therefore, to reach a particular conclusion, in our case concerning the degree of subtle myocardial injury, a specific summarizing index that would synthesize the effects of individual components is necessary. The calculation method of such an index can be implemented in different ways. However, it must always include such sequential steps as theoretical justification of the composite index for the particular task selection of data adequate to the problem at hand analysis of this data, including its normalization, using methods of multivariate statistics selection of the informative private indicators (including the exclusion of correlated parameters) and finally an actual construction of the composite index through the aggregation of private indicators. As was shown above, we have fulfilled all those steps. At the same time, it is crucial to consider that in addition to the composite index, various partial indicators are the most informative for detecting subtle changes in various clinical scenarios.

Usually, those are modern electrocardiographic indexes with a common pathophysiological basis. All of them assess the electrical homogeneity of the myocardium through different means, as the more heterogeneous the myocardium is from an electrical point of view (the higher the dispersion of the generated transmembrane action potentials in amplitude and length), the higher the likelihood of serious cardiovascular events. In our study, such a highly informative modern electrocardiographic index was the T-wave SVD.

The SVD of T-wave represents the complexity of ventricular repolarization. One major spatial component (eigenvector) can be identified when repolarization is uniform, as in normal individuals. Conversely, when the repolarization pattern becomes fragmented, the relative value of the smaller vectors increases proportionally. Such an approach allows a comparison between the morphology of the T-wave across the 12 leads and the quantification of T-wave abnormalities in an observer-independent way (Clarke et al., 2020).

A key element in conducting our original research was the integration of an advanced Hybrid Cloud Environment for Telerehabilitation (HCET) (Malakhov, 2024; Malakhov and Semykopna, 2024), developed at the Glushkov Institute of Cybernetics of the National Academy of Sciences of Ukraine. Building on the concept of Research and Development Workstation Environments (RDWEs) (Palagin et al., 2018), HCET provides a robust, ontology-driven, service-oriented architecture that supports each stage of clinical research: from data collection and secure storage to real-time analysis and interpretation of results.

The HCET infrastructure (Malakhov, 2024) merges distributed hardware resources, specialized software platforms, and dedicated telemedicine modules, enabling seamless collaboration among multidisciplinary teams. Its three-layer model – Infrastructure-as-a-Service (IaaS), Platform-as-a-Service (PaaS), and Software-as-a-Service (SaaS) – ensures the flexible allocation and on-demand scaling of computational resources. At the core of the infrastructure is a high-performance server (HP ProLiant DL380p Gen8) running Ubuntu 22.04.3 LTS, configured with virtualization tools (KVM, LibVirt) to host multiple virtualized operating systems. This setup supports data-intensive applications and fosters reliable data processing pipelines.

Specifically, HCET facilitated several key steps in this study of ECG changes in severe COVID-19 patients:

Secure Data Acquisition and Management. Patient ECG data, along with relevant clinical parameters (e.g., NEWS scores), were collected in the ICU setting and securely transmitted to the HCET server. The platform’s integrated Electronic Health Record (EHR) managing module ensured proper storage, encryption, and retrieval of sensitive information, adhering to ethical and legal requirements for patient confidentiality.

Advanced Analytical Tool Integration. The HCET environment included support for Statistica 12.0 software, which was pivotal for our repeated-measures ANOVA, cluster analysis, and decision tree modeling. By leveraging the platform’s PaaS capabilities, researchers could seamlessly install, configure, and run Statistica within multiple virtual operating systems, optimizing both performance and collaborative workflows.

Interactive remote collaboration. Telemedicine components within HCET, such as the “Physician’s Digital Workplace” and “Patient’s Digital Cabinet,” enabled continuous interaction between clinicians, biomedical engineers, data scientists, and other stakeholders. This not only streamlined the process of updating patient records but also allowed real-time discussion of ECG findings, NEWS scores, and relevant biomarkers across geographically dispersed locations.

Machine Learning and Decision Support. Building on the RDWE principles, the cognitive subsystem of HCET (referred to as the information-analytical subsystem) supported iterative model development. Automated interactive systems—such as OntoChatGPT (Palagin et al., 2023)—facilitated the semantic and contextual analysis of text documents. Researchers also used the specialized MedRehabBot (Palagin et al., 2024) services to manage domain-specific ontologies, aiding in the consistent labeling and categorization of ECG parameters. This approach improved the accuracy of clustering methods (Kaverinskiy et al., 2024a; Kaverinskiy et al., 2024b) used to pinpoint subtle myocardial injury and strengthened the decision-tree models predicting COVID-19 mortality.

Scalability and future directions. As additional clinical evidence accumulates, HCET’s hybrid cloud setup ensures that resources can be dynamically scaled to accommodate larger patient cohorts, additional ECG recordings, and refined machine learning algorithms. Such scalability supports ongoing research into minor ECG changes, fostering deeper insights into myocardial injury, especially in post-COVID-19 care.

In essence, integrating the HCET platform into our experimental design bolstered every phase of this original research, from data stewardship to advanced statistical analyses. By uniting telemedicine, digital health, and cloud-based data processing services, HCET exemplifies how innovative hybrid cloud solutions can effectively support cutting-edge studies in physical medicine, rehabilitation, and beyond.

This work is the first study to assess minor electrocardiogram changes using the original scaling method in patients with COVID-19.

We reiterate that patients with unstable comorbid conditions and/or significant resting ECG abnormalities, as defined by Minnesota coding, were excluded from the study. This step was taken to ensure that any dynamic ECG changes observed would be attributed to COVID-19 rather than other underlying diseases. These findings underscore that our results may not be fully generalizable to individuals entirely free of comorbidities. Future research should expand recruitment to patients without these comorbidities to verify whether U-score based ECG changes exhibit similar predictive value.

Limitations of the study are the following: Firstly, the number of patients is relatively small. Secondly, no comparison of minor ECG changes with the levels of biomarkers of myocardial damage and inflammation was performed. Finally, the prognostic value of detected ECG changes regarding long-term COVID complications has yet to be analyzed.

Further larger-scale studies are planned to confirm and clarify the results.

## 5 Conclusion

The suggested ECG and HRV scaling method allow for registering and analyzing minor electrocardiogram changes during treatment. Modern ECG parameters used for advanced ECG analysis were the most informative. Contrary to this outline, the ECG analysis must be more informative for this task.

Two subgroups were identified that differed significantly in the severity of COVID-19 and the integral indicator of the cardiovascular system at the beginning of treatment. At the end of treatment, differences between subgroups remained. In the severe subgroup, there were 50 percent of deaths.

A comparison of potential predictors of mortality showed that at the beginning of treatment, there were no significant differences in vital signs between those who survived and those who died. In our study, the average age in the group of deceased patients was slightly higher, and the SAPS II score was not associated with the treatment outcome. A set of ECG parameters significantly associated with treatment outcome and may be predictors of treatment outcome were identified.

In addition to the U-score based composite index, partial indicators are the most informative for detecting subtle changes in various clinical scenarios, such as treatment outcome prediction. A decision tree for the survival classification of patients with COVID-19 was built based on the partial ECG parameters and NEWS score.

The obtained results allow us to create a combined decision rule that includes both the well-known NEWS-score and AI-based analysis of subtle ECG changes. Such a combined decision rule should have the highest possible predictive accuracy for COVID-19 Treatment Outcome. Further large-scale studies are needed to confirm these findings.

An important future direction involves utilizing innovative informatics infrastructures, such as the Hybrid Cloud Environment for Telerehabilitation (Malakhov, 2024; Malakhov and Semykopna, 2024), to manage and analyze large ECG datasets alongside clinical metrics. By delivering on-demand computational resources within an integrated platform, HCET enhances the scalability of machine learning pipelines for real-time ECG data processing.

## Data Availability

The raw data supporting the conclusions of this article will be made available by the authors, without undue reservation.
